# Desquamation of the subpleural lung parenchyma caused by empyema after pulmonary embolism: A case report

**DOI:** 10.1002/rcr2.1008

**Published:** 2022-07-18

**Authors:** Takafumi Iguchi, Shinjiro Mizuguchi, Chung Kyukwang, Ryu Nakajima, Makoto Takahama

**Affiliations:** ^1^ Department of General Thoracic Surgery Osaka City General Hospital Osaka Japan

**Keywords:** bronchopleural fistula, empyema, pulmonary embolism, subpleural lung parenchyma, thoracostomy

## Abstract

Subpleural peripheral lung regions are mainly nourished by pulmonary arteries. Herein, we report a case in which pleural infection after pulmonary embolism caused circulation failure in the subpleural lung parenchyma (SLP) and massive desquamation of the SLP.

## Graphical abstract

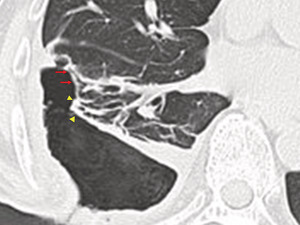

## CLINICAL IMAGE

**FIGURE 1:**
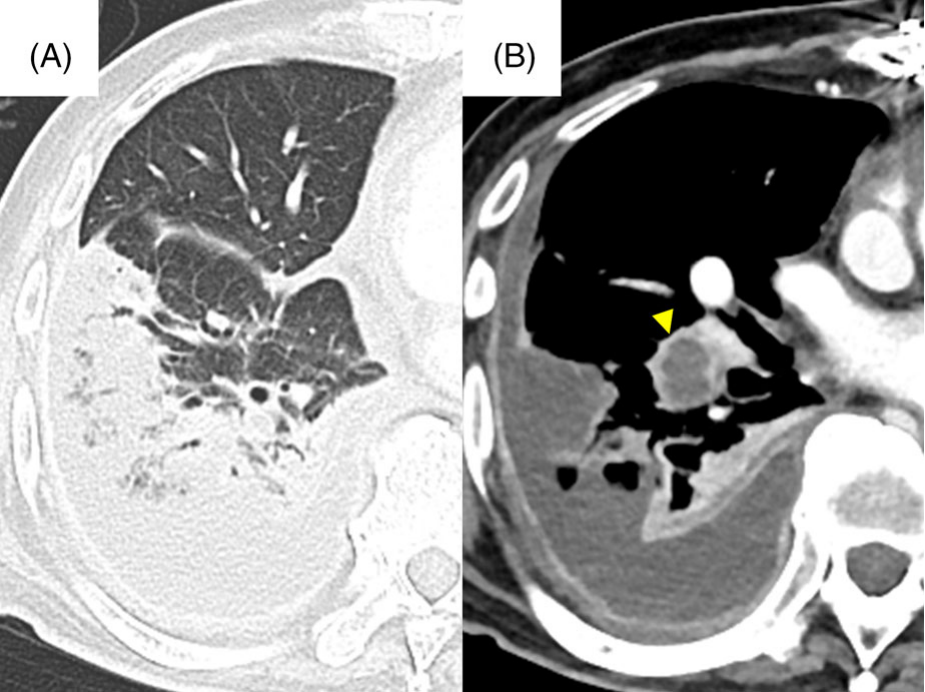
Computed tomography (CT) of the chest at 2 weeks after the embolectomy. (A) Chest CT reveals peripheral lung consolidation extending over the right lower lobe and the right pleural effusion. (B) Contrast CT shows that a contrast defect of the right basal pulmonary artery (i.e., embolus, indicated by arrowhead) remains.

**FIGURE 2:**
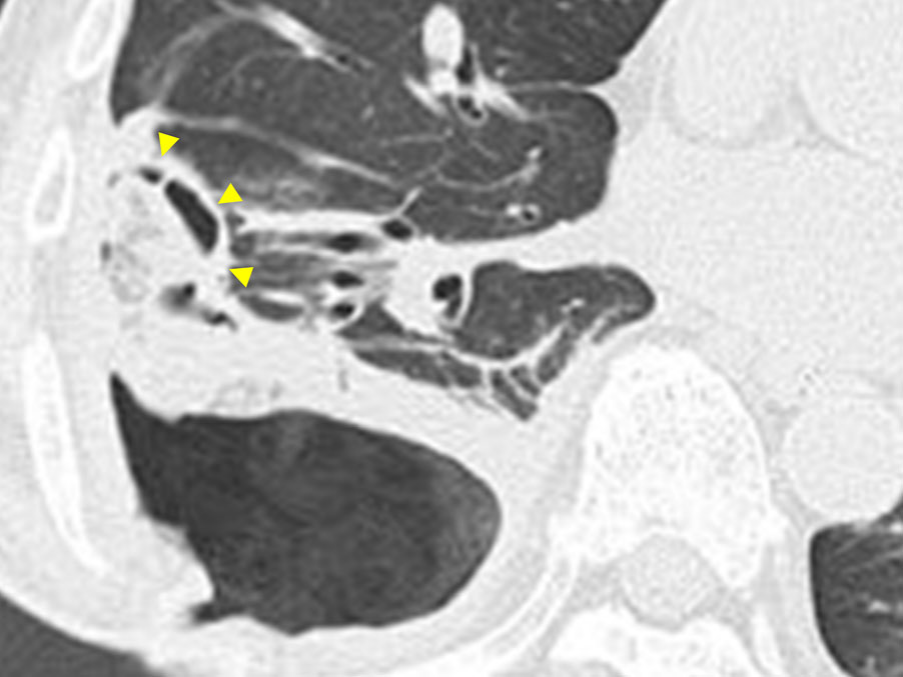
Computed tomography of the chest at 1 month after the thoracostomy. The subpleural lung parenchyma was necrosed along the subpleural line (arrowheads).

**FIGURE 3:**
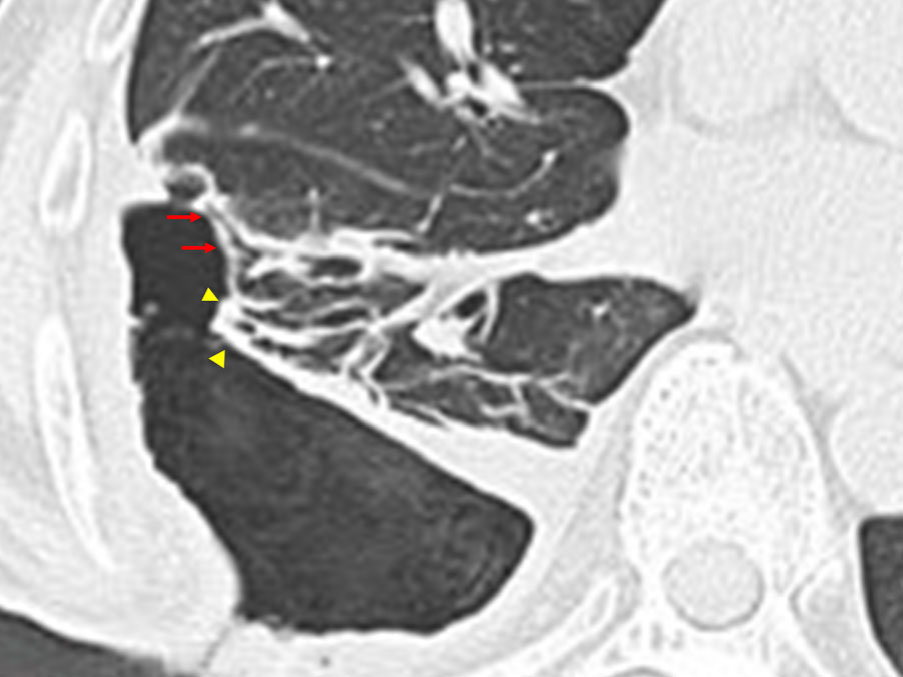
Computed tomography of the chest at 1.5 months after the thoracostomy. The subpleural lung parenchyma in the right B8 (arrows) and B9 (arrowheads) regions is completely desquamated and numerous bronchial fistulas have appeared.

**FIGURE 4:**
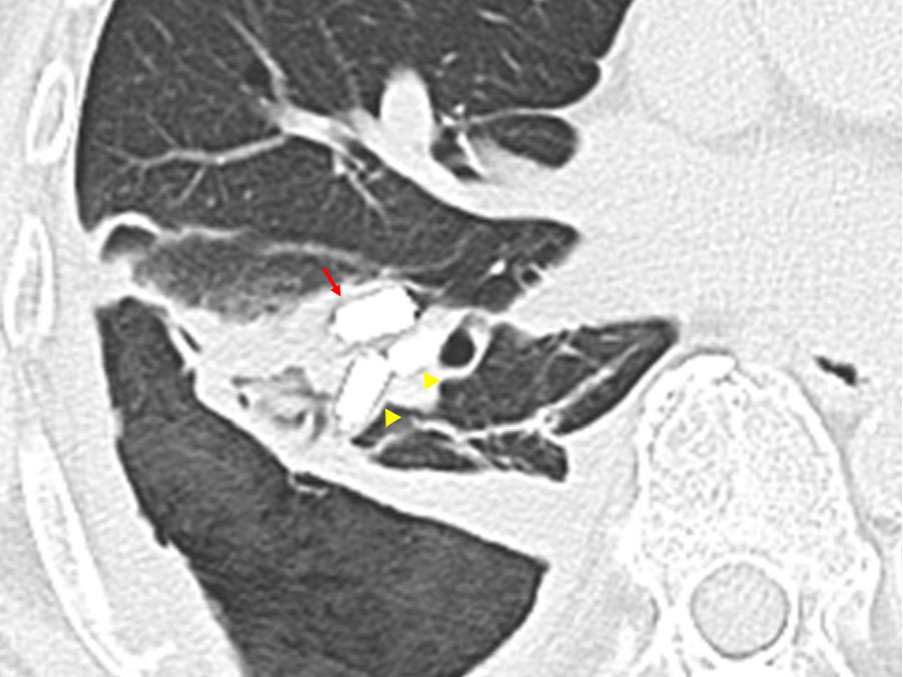
Computed tomography of the chest after the endobronchial occlusion showing appropriate deployment of silicone spigots at right B8 (arrow) and B9 (arrowheads) and the absence of the bronchial fistulas.

A 73‐year‐old male underwent right pulmonary embolectomy for septic pulmonary embolism with right‐sided endocarditis. Although the postoperative course was uneventful for 2 weeks, the deep part of the right basal pulmonary embolus remained (Figure 1). Two months after the operation, aspiration pneumonia was diagnosed, progressing to right empyema. Diagnostic thoracoscopy findings showed that the surface of the right lower lobe was covered with necrotic tissue, and an open‐window thoracostomy was performed. One month after the thoracostomy, bronchial fistulas were detected (Figure 2). Two weeks later, the air leak increased causing a hoarse voice, and the subpleural lung parenchyma (SLP) was extensively desquamated (Figure 3). Endobronchial occlusion with silicon spigots was performed, providing significant reduction of the air leak (Figure 4). The cavity was still draining 6 months after the thoracostomy. The mechanism of the SLP desquamation in the present case was related to the discrepancy between the areas nourished by the pulmonary arteries (PAs) and the bronchial arteries (BAs). The BAs provide nourishment to the supporting structures of the lungs central to the terminal bronchiole, but feeding of the SLP mainly depends on the PAs.^1^ Additional pleural infection with persistent PA occlusion caused circulation failure in the SLP with massive desquamation.

## AUTHOR CONTRIBUTION

Takafumi Iguchi and Shinjiro Mizuguchi were involved in the conception or design of the work and the acquisition, analysis or interpretation of data. Chung Kyukwang and Ryu Nakajima drafted the work or revised it critically for important intellectual content. Makoto Takahama was involved in the final approval of the version to be published.

## CONFLICT OF INTEREST

None declared.

## ETHICS STATEMENT

The authors declare that appropriate written informed consent was obtained for the publication of this manuscript and accompanying images.

## Data Availability

The data that support the findings of this study are available from the corresponding author upon reasonable request.
